# Keeping up with the Joneses: Interpersonal Prediction Errors and the Correlation of Behavior in a Tandem Sequential Choice Task

**DOI:** 10.1371/journal.pcbi.1003275

**Published:** 2013-10-24

**Authors:** Terry Lohrenz, Meghana Bhatt, Nathan Apple, P. Read Montague

**Affiliations:** 1 Virginia Tech Carilion Research Institute, Roanoke, Virginia, United States of America; 2 The Wellcome Trust Centre for Neuroimaging, University College London, London, United Kingdom; University of Oxford, United Kingdom

## Abstract

In many settings, copying, learning from or assigning value to group behavior is rational because such behavior can often act as a proxy for valuable returns. However, such herd behavior can also be pathologically misleading by coaxing individuals into behaviors that are otherwise irrational and it may be one source of the irrational behaviors underlying market bubbles and crashes. Using a two-person tandem investment game, we sought to examine the neural and behavioral responses of herd instincts in situations stripped of the incentive to be influenced by the choices of one's partner. We show that the investments of the two subjects correlate over time if they are made aware of their partner's choices even though these choices have no impact on either player's earnings. We computed an “interpersonal prediction error”, the difference between the investment decisions of the two subjects after each choice. BOLD responses in the striatum, implicated in valuation and action selection, were highly correlated with this interpersonal prediction error. The revelation of the partner's investment occurred after all useful information about the market had already been revealed. This effect was confirmed in two separate experiments where the impact of the time of revelation of the partner's choice was tested at 2 seconds and 6 seconds after a subject's choice; however, the effect was absent in a control condition with a computer partner. These findings strongly support the existence of mechanisms that drive correlated behavior even in contexts where there is no explicit advantage to do so.

## Author Summary

In this study we examine the neural substrates of inter-personal error signals on behavior in an investment task using real historical markets. We show that behaviorally, subjects correlate their investments, despite the fact that another trader has no extra information about how the market may move. These behavioral results are supported by neural data showing large, parametric responses in brain areas related to reward and learning when information about another trader's behavior is revealed, even though this occurs after all useful information about the market has already been shown. These results promise to elucidate some of the subconscious processes that guide people to correlate their behavior in markets and other group environments.

## Introduction

Humans learn a range of information from one another [1] and show a particular sensitivity to the influence of group behavior [2]. The ultimate evolutionary origins of these behaviors and their dependence on other relevant variables raise broad-ranging questions [3]–[6] however, they also invite important but narrower questions about the human propensity to assign value to the behavior of others even when there exists no external incentive to do so. Such assignments can reasonably be considered irrational because they explicitly violate external incentive structures. It has been suggested that this propensity to ‘follow-the-crowd’ – even in the face of information that suggests otherwise – is the basis of a range of herding behaviors displayed by humans interacting through markets including both bubbles and crashes [7]–[11]. One hypothesis for the origin of this class of ‘believe-the-group’ irrationalities is that while long ago group behavior tended to be a good proxy for value, the complexities of modern life, and especially modern markets, subvert this tendency, producing unpredictable behaviors in market settings.

**Figure 1:**
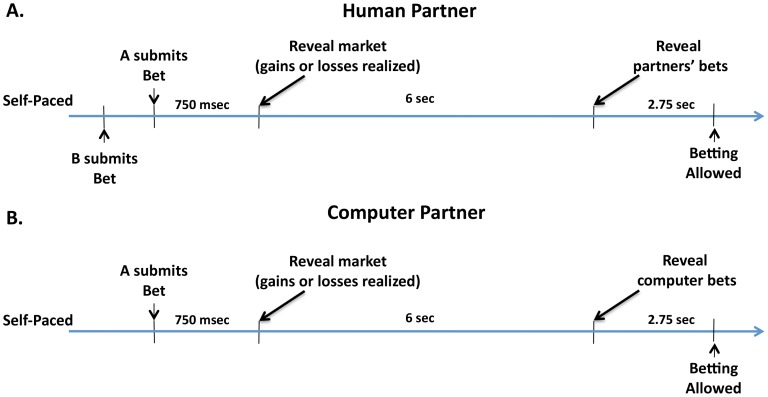
Schematic experiment timeline. **A.** Two subjects simultaneously played a sequential investment task. After receiving market information each player privately submitted their own bet. After a delay the players' bets were simultaneously revealed to the other player (Exp 1, 2 sec delay, 68 subjects; Exp 2, 6 sec delay, 24 subjects), and then another round began. **B.** In experiment 2 subjects were told and in fact played a computer partner which chose investments randomly.

We used a tandem (two-person) sequential choice experiment, framed as a market investment task, to test the degree to which neural and/or behavioral responses change depending solely on the behavior of one's partner, and whether they do so in the absence of incentives. The task asks a subject to invest some fraction (from 0 to 1) of their total holdings, shows the change in the market value which controls gains and losses, and later shows the fraction invested by their partner (Figure 1). The partner's investment has no bearing on the payoff of the subject or on the market's future movements. In addition to this tandem task we included a control condition in which subjects played in tandem with a computer that chose its investments randomly (uniformly over [0,1]). In this control condition, subjects were informed that the other “investor” was a computer and that its choices were random. This experiment asks two empirical questions. (1) Does a subject change their behavior based on the difference with their partner's choice (Jones)? (2) How does the brain respond to the difference between the subject's investment level and their partners? We repeated the experiment twice and varied the time at which the partner's choice was revealed (2 seconds and 6 seconds after the subject's choice).

In this task, there is no incentive for the answer to either question to be yes; however, a positive answer to either suggests that group behavior is deemed valuable by brain and behavior even in the absence of external economic incentives. The striatum is well known for encoding “prediction error” signals that aid humans and other animals learn the value of various stimuli and actions; therefore, we hypothesized that the “interpersonal prediction error”, i.e. the difference between the partner's and the subject's own bet (henceforth referred to as Jones), would (a) correlate with activation in the striatum and (b) correlate with the bet in the next round of play. Hypothesis (a) is based on the idea that the subject's brain assumes that this difference with the partner's bet is an informative error signal. Hypothesis (b) – the idea that this difference would correlate with a tendency to adjust ones behavior toward that of the other investor – suggests one bias that would encourage irrational herding behavior.

The setup for our tandem investment task and our framing of the behavior in terms that inform our notion of irrational herding behavior is also supported by economic ideas. Economists have laid out the theory of information cascades – situations where *rational* agents disregard their private signals and follow the choices of others [9], [12], [13] ‘as though’ the others have different or better information. This tendency to herd is also thought to play a role in more complicated situations, such as financial markets, where the phenomenon may lead to bubbles and crashes [14].

Recently neuroscientists have begun to explore the neural underpinnings of social learning [15]–[23]. We extend these results to consider the effects of others' past investment behavior on subsequent investment behavior when the risk parameters of the underlying market are fundamentally unknown. We hypothesized that modulation of the error signals in the ventral striatum would reflect the influence of social information on investment behavior.

## Results

In order to test the hypothesis that people's investment behavior is affected by social information, and to probe the neural substrates of this influence, we employed fMRI and two human versions and one control condition of a “tandem” implementation of an investment game previously used to probe intrapersonal fictive errors (the difference between the actual received reward, and the best possible outcome a subject might have achieved) [24], [25]. Figure 1 gives a schematic outline of the tasks. In the human conditions two subjects (who knew that there was another person playing but did not meet) played the investment game simultaneously while being scanned. In the investment game, both subjects were endowed with $20, and then each had to decide what percentage of their endowment to risk in the “market” (the markets were taken from actual historical markets. See Text S1 for details). After each person lodged their asset allocation (their “bet”), the next market outcome was revealed, the portfolio value and percentage gained or lost was updated, and after a short delay (2 sec in the first version, and 6 sec in the second) a pair of red arrows representing the other player's investment level percentage appeared on the slider bar. After another short delay the process was repeated. The choices of the players had no direct influence on future market fluctuations, and the choices of one player had no direct influence on the payoffs of the other player. In the computer control condition the subject was told they were playing a computer partner that chose randomly; the delay between the market revelation and the revelation of the computer choice was 6 sec. We examined the behavior from all three experiments, but focused on the imaging from the 6 sec. human and computer control conditions.

### Behavior

**Figure 2:**
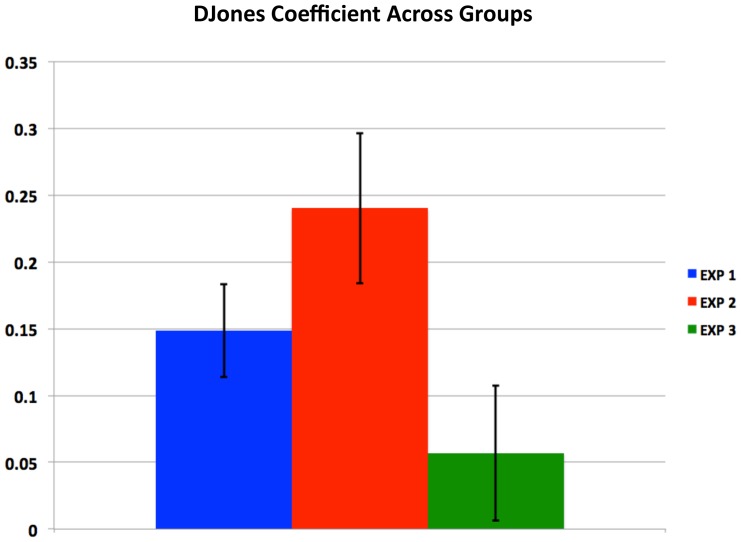
Behavioral analyses of the sequential investment task reveals influence of other player's investment. Multiple regression analysis shows that a player's next bet was influenced significantly by the difference between the human partner's bet and their own bet (DJONES). This was true whether the result was lodged at 2 seconds, Experiment 1, or 6 seconds, Experiment 2, after the revelation of the market return. The influence of DJONES was not significant in the computer control Experiment 3.

To examine differences among the three versions of the experiment we performed a mixed-effects linear regression separating the three groups (2 sec human, N = 68; 6 sec human, N = 24; 6 sec computer control, N = 24; see Tables S1, S2, S3 for demographic information) using indicator functions for the three groups (interacting with all of the variables of interest). The dependent variable was the normalized investment. The independent variables in the regression were a constant, the normalized previous bet, the previous market return (MKT), and a variable we call DJONES, equal to the difference between the other subject's investment and the subject's investment. Here we focus on the regression coefficient of DJONES (Figure 2). The coefficients from the 2 sec and 6 sec human experiments are both significantly greater than zero, and the coefficient in the computer control condition is not significantly different than zero. There is also a significant difference between the human 6 sec condition, and the computer control condition. See Text S1 for more regression details, and Table S4 and Table S5 for complete regression tables.

### Neuroimaging

**Figure 3:**
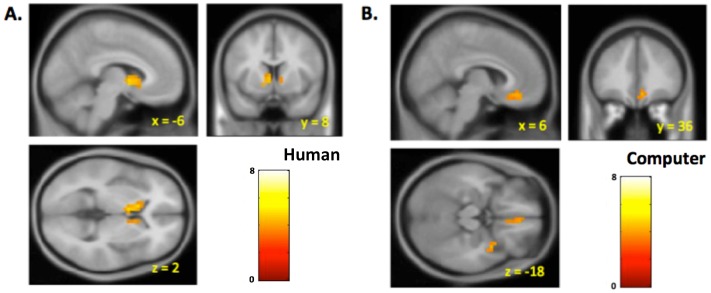
Striatum shows a parametric response to interpersonal error with human partner. **A.** When subjects play the human partner in Experiment 2, the striatum shows a parametric correlation with the interpersonal error, the difference between the other player's bet and the player's bet (DJONES). **B.** When subjects play a computer partner there is no significant activation associated to DJONES in the striatum. Both figures p<001, cluster size > = 5, n = 24. The Experiment 2 (human partner) striatal activation survives a small volume correction, as does the contrast human>computer.

To investigate the neural underpinnings of these signals we constructed a regression model for the imaging data using regressors suggested by behavioral model (see Supporting Information for details). We limited our investigation of the neural data to the 6 sec human and computer control experiments. Specifically we included a parametric regressor for DJONES at the reveal of the other person's investment, and a parametric regressor for MKT at the time of the revelation of the market return to the subject. Figure 3A shows the activation corresponding to the DJONES regressor in the human condition while 3B shows the activation in the computer control (both N = 24; both displayed with p<.001 uncorrected, cluster size > = 5). Note that there were no regions of significant negative correlation. See Figure S1 and Figure S2 for regression tables and glass brains. In the human condition, this activation survived a small volume correction for multiple comparisons over an ROI consisting of 5 mm radius balls centered on bilateral caudate/putamen voxels taken from peak activations in [24]. (See Figure S3 for mask). Additionally, the comparison (two-sample t-test) of DJONES across the human and computer conditions survived a similar small volume correction yielding voxels in left caudate (Figure S4). Activation tables for both small volume corrections are in Figure S5.

While not our main focus, it is worth noting that the MKT regressor also produced, in both human and control conditions, robust activation in the striatum (Figure S6). Figure S7 shows a conjunction/disjunction analysis of the MKT and DJONES activation at the p<.001 and p<.05 levels in the human condition.

We were also interested in the possible differences between the neural and behavioral effects of the variables obtained by splitting DJONES into its positive and negative parts (e.g. POSDJONES = max(DJONES , 0); see Text S1 for details). We find a significant difference in the behavioral regression coefficients, with the coefficient of the negative part of DJONES being larger in absolute value (Table S3). Neurally, however, we find no difference between the two conditions (Figure S8).

**Figure 4:**
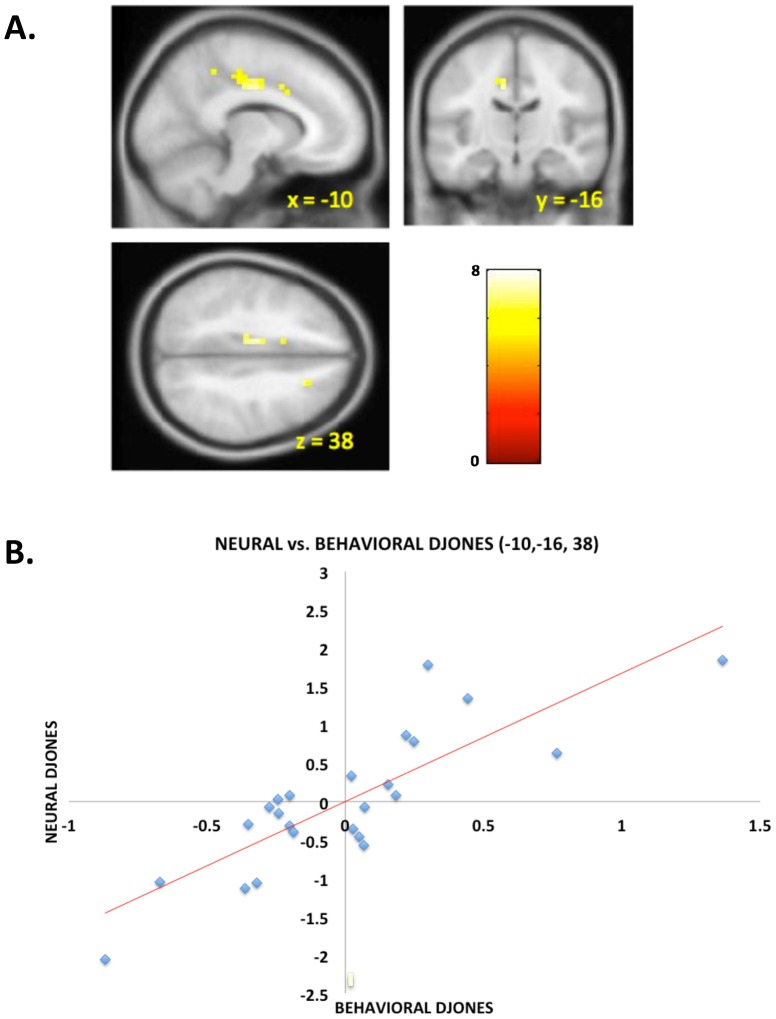
Neural interpersonal error sensitivity covaries across subjects with behavioral influence of interpersonal error in middle cingulate. **A.** Across subject regression of the beta maps from the neural DJONES regressor against the behavioral DJONES coefficients revealed a significant (p<.05, whole – brain FWE corrected) linear relationship in middle cingulate. **B.** Plot of individual values of neural DJONES from peak voxel in middle cingulate (MNI [−10,−16,38]) versus individual behavioral DJONES coefficients (mean adjusted).

Finally, we wanted to investigate the relationship between the neural correlates of DJONES and the individual behavioral regression coefficients of DJONES. Figure 4A shows the middle cingulate region for which the individual neural DJONES responses are significantly positively linearly related to the individual behavioral DJONES coefficients (*p*<.05, FWE whole-brain corrected; behavioral coefficients from individual subject regressions. See Text S1 for details.). Figure 4B shows (for illustrative purposes only) a plot of the neural coefficients against the (mean adjusted) behavioral coefficients.

## Discussion

Using a tandem sequential investment task we show that when subjects play a human partner the inter-personal fictive error guides behavior (subjects' next bet) and correlates with a robust neural signature in the striatum. These findings are significant because the partner's choice is revealed after the subject's monetary outcome is revealed and the partner's choice has no bearing on the payoff to the subject. Despite these facts, the inter-personal fictive error still influences the subject's behavior on their next bet, correlates with a robust and parametric neural signature in an important reward processing structure, and depends on whether the partner is a human. Specifically, if humans play a computer partner expressing random investments on each trial this same inter-agent fictive error term has no behavioral impact on the next bet and has no significant neural correlate in the striatum.

Our results are for the most part are consonant with the results of previous studies of social influence [15]–[23] that show neural responses to and behavioral influences of the choices of others. However, there are several key differences that allow us to expand on these results.

First, the timing of private and social outcome revelations was significantly different in this design. Here, information about the market is revealed first, giving the subject all the information relevant to their payoff, and then the social signal from the partner is revealed. Second, our design is parametric in the choices and outcomes. Our design thus allows us to show that the striatal response and immediate subsequent behavior is fully parametrically influenced by both the market return signal and the interpersonal error signal. Additionally, we see a behavioral asymmetry in the effect of the partner's investment between the outcome where the partner invested more than the subject versus the case where the partner invested less. Subjects adjusted their subsequent investment more when their partner invested less than they did on the previous trial as though they were fleeing their own over-exuberance on that trial. Finally, Burke et al. [17] show that ventral striatum activation to social information covaries with behavioral sensitivity to herd information. We do not see this in our experiment. Rather, we see that neural activation to DJONES in middle cingulate cortex covaries positively with behavioral sensitivity to DJONES. One possible explanation for this correlation is suggested by two studies. Kishida et al. [26] found that athletes showed increased middle cingulate activity when imagining themselves playing their own sport as opposed to a different sport. Further, they saw the same result in subjects when they took a first, as opposed to third person perspective when imagining a sports scene. On the other hand, Chiu et al. [27] found decreased activity during the “self” phase of the trust game in the middle cingulate in autistic subjects. The effect covaried with symptom severity. These results suggest that this area is key for identifying with conspecifics, pointing to a hypothesis that neural sensitivity in middle cingulate to the DJONES signal is dependent on the tendency of a subject to identify with the other investor. This hypothesis is also supported behaviorally by the findings of Burke et al. [17] showing that herding behavior is more pronounced when investing alongside human conspecifics as opposed to non-human primates, as well as by the absence of a DJONES effect in our control condition.

Our results suggest that the difference between the partner's investment and the subject's investment can be viewed as an error signal that guides behavior, rather than as simply an add-on affective response. The affective system has long been considered a necessary component effective decision-making [28] whose function can be seen as “ecologically rational” [29]. Neural signals correlated with affect may then be reinterpreted as error signals [30]. For example, much of the early work on anterior insula focused on emotions such as pain and disgust. [31], [32] Recently, however, the function of the anterior insula has been recast in the language of error signals [29], whereby activation in the insula is regarded as signaling a variance prediction error. Here our focus is on the striatum, but the idea is similar. Indeed multiple works [17], [23], [33]–[35] suggest that socially construed reward signals should appear in the striatum just as other control signals do. In this light, the results of this paper strongly suggest that we view the activation in the striatum not only as a hedonic signal, but also as a control signal.

Correlation is a property that is vitally important in asset management: in order to maximize return with a minimum of risk an investment manager must know the correlation of the assets under management [36]. Our ancestors living in small groups were not “asset managers”, but it is likely the members of the group correlated their activities in an optimal way, an activity that would require the brain to track and control individual correlations.

Finally these results provide biological evidence that standard theories of investment behavior that are variations on the Markowitz model [37] miss a fundamental driver of behavior by failing to account for the behavior of other investors. The response of the striatum to the Jones variable suggests that tendency to correlate actions is deeply rooted with potential evolutionary drivers. This lends weight to the “behavioral finance” approach espoused by Shiller and others [10], [38], [39].

In summary, previous work shows that the comparison of personal results to the results of another modulates neural activity. Our results further show that the comparison of the personal result to the outcome of the other person can be put in the context of an error signal, the interpersonal fictive error, which controls behavior and has a robust neural signature. Social comparison can thus be construed not merely as a possibly unseemly manifestation of envy, but rather as a potentially useful learning signal.

## Materials and Methods

### Ethics statement

Informed consent was obtained for all research involving human participants, and all clinical investigation was conducted according to the principles expressed in the Declaration of Helsinki. All procedures were approved by the Institutional Review Board of the Baylor College of Medicine, or the Institutional Review Board of Virginia Tech.

### Participants

Experiment 1: 76 participants were recruited and 74 scanned in accordance with a protocol approved by the Baylor College of medicine IRB. In the two behavioral only subjects the log files of the experiment were incomplete, leaving unusable data; in two scanned subjects the experiment terminated prematurely; in 4 other scanned subjects the functional images were unusable, leaving 68 subjects with both behavioral and imaging data. Table S1 summarizes the demographic information of these 68 subjects. All data mentioned in the text and supplementary information referring to the first experiment refers to the behavioral data only of these 68 subjects. Experiments 2 and 3: 49 participants (24 for the human condition and 25 for the computer control condition) were recruited and 49 scanned in accordance with a protocol approved by the Virginia Tech IRB. One subject's scanning session terminated prematurely in the control cohort leaving 24 subjects. All data mentioned in the text and SOM referring to the second experiment refers to these subjects. Table S2 summarizes the demographic information of these subjects.

### Task

Participants arrived at the lab, were consented, and then read task instructions. In the versions with human partners the partners did not meet. After they were loaded in scanner, the task began. Each subject participated in 10 markets in a random order. There were two groups of markets, A and B (originally described and used in Lohrenz et al., 2007). 30 subjects saw group A, and 41 subjects saw group B. After seeing initial market data, a participant selected an investment level (0% to 100% in increments of 10%) using one button box (shown on a slider bar on the screen) and submitted the decision using the other button box. In the human partner versions the next market result appeared 750 ms after the later of the two partners' choice was submitted. In the computer partner version the result was displayed 6 seconds later. 2 or 6 seconds later (depending on the experimental cohort, 1 or 2,3) the other partner's choice, was displayed by showing two red arrows on either side of the slider bar showing the level person's investment. This was repeated 20 times per market, for a grand total of 200 decisions.

### fMRI data

#### Data acquisition

Imaging data were collected at Virginia Tech Human Neuroimaging Lab using 3-tesla Siemens TRIO scanners. Initial high-resolution T1-weighted scans were acquired using an MP-Rage sequence. Functional images were acquired with TR = 2000 ms and TE = 25 ms. 37- mm slices were acquired 30 degrees to the anteroposterior commissural line, yielding functional voxels that were 3.4 mm×3.4 mm×4 mm.

#### Preprocessing

All data were preprocessed using standard SPM8 algorithms (http://fil.ion.ucl.ac.uk/spm, ). Functional images were motion corrected using a six-parameter rigid-body transformation to the first functional scan. The mean functional images for each respective subject were then co-registered using a twelve-parameter affine transformation to the subject's high resolution T1 structural scan. The subject T1 was segmented into gray and white matter and then normalized to the MNI template, and the functional images normalized to the template, with resampled 4×4×4 mm functional voxels. Functional images were smoothed spatially using a 8 mm Gaussian kernel.

#### Analysis

All functional data were high-pass (128 sec) filtered. The AR 1 structure option was used in SPM8. For each subject a design matrix was constructed using canonical events (each event was punctate, and convolved with the standard hemodynamic response function (HRF) in SPM8; see Text S1 for full details). Of particular interest were the events REVEAL and JONES REVEAL. REVEAL was the event where the new market trace was revealed to each subject (see Figure 1). JONES REVEAL was the event where the investment of the partner was revealed (see Figure 1). One additional regressor was formed by parametrically modulating REVEAL with MKT (the market return – see Text S1 for full details). A second additional regressor was formed by parametrically modulating JONESREVEAL with DJONES (the difference between the partner's investment and the subject's investment). The beta images for the MKT and DJONES from the first-level analysis were entered into a second-level t-test for the analyses presented in Figure 3.

### Behavioral analysis

Subject's behavioral data were analyzed in R (package nlme) [40], [41] (see Text S1 for full details).

## Supporting Information

Figure S1 Imaging table and glass brain for DJONES regressor (p<.001, uncorrected k ≥5, n = 24) in experiment 2 ( 6 sec. human partner).
(TIF)

Figure S2 Imaging table and glass brain for DJONES regressor (p<.001, uncorrected k ≥5, n = 24) in experiment 3 ( 6 sec. computer control partner).
(TIF)

Figure S3 Images of mask used in small volume corrections. Regions of L/R caudate and L/R venral striatum formed by the union of 5 mm radius balls centered on peak activation coordinates from Lohrenz et al. 2007 [24] (Caudate: (-8, 8, 4), (8, 12, 4). Ventral Striatum: (-16, 8,-12), )16, 12,-12). MNI coordinates).
(TIF)

Figure S4 Two-sample t-test image of the comparison human>computer for the DJONES regressor (p<.005. cluster size > = 5, uncorrected, n1 = 24, n2 = 24).
(TIF)

Figure S5 Small volume correction statistics for: A. the DJONES regressor in experiment 2 over the region of interest displayed in Figure S3; B the comparison human > computer (experiment 2 > experiment 3) for the DJONES regressor.
(TIF)

Figure S6 Left: MKT regressor in experiment 2, human partner (p<.001, cluster size > = 5, uncorrected, n = 24); Right: MKT regressor in experiment 3, computer control partner (p<.001, cluster size > = 5, uncorrected, n = 24).
(TIF)

Figure S7 Conjunction/disjunction images for the DJONES and MKT regressors in experiment 2 (human partner). Left: masks created using p<.001, cluster size > = 3, uncorrected, n = 24; Right: masks created using p<.05, cluster size > = 3, uncorrected, n = 24.
(TIF)

Figure S8 Left: Thresholded t-map of the regressor POSDJONES in experiment 2 (p<.001, cluster size > = 5, uncorrected, n = 24; nb: positive correlation). Right: Thresholded t-map of the regressor NEGDJONES in experiment 2 (p<.001, cluster size > = 5, uncorrected, n = 24; note: negative correlation).
(TIF)

Figure S9 Thresholded t-maps for the within-subject contrast NEGDJONES+POSDJONES. Left, positive correlation, right negative correlation (p<.05, cs > = 5, uncorrected, n = 24).
(TIF)

Table S1 Experiment 1 (2 sec human partner experiment) demographic information (N = 68).
(TIF)

Table S2 Experiment 2 (6 sec human partner experiment) demographic information (N = 24).
(TIF)

Table S3 Experiment 3 (6 sec computer control experiment) demographic information (N = 24).
(TIF)

Table S4 Regression fixed-effect coefficient estimates for the grouped three-experiment behavior.
(TIF)

Table S5 Contrast estimates for the grouped three-experiment behavior.
(TIF)

Table S6 Behavioral regression fixed-effect coefficient estimates for experiment 2 (6 sec human partner for the POSDJONES/NEGDJONES model; n = 24).
(TIF)

Table S7 Contrast estimate for experiment 2 (6 sec human partner for the POSDJONES/NEGDJONES model; n = 24).
(TIF)

Text S1 Supplementary information on task and analysis.
(DOCX)
