# Supplementary figures and images for: Keeping up with the Joneses: Interpersonal Prediction Errors and the Correlation of Behavior in a Tandem Sequential Choice Task
Source: PLoS Comput Biol. 2013 Oct 24;9(10):e1003275. doi: 10.1371/journal.pcbi.1003275 (PMC3812045; doi:10.1371/journal.pcbi.1003275)

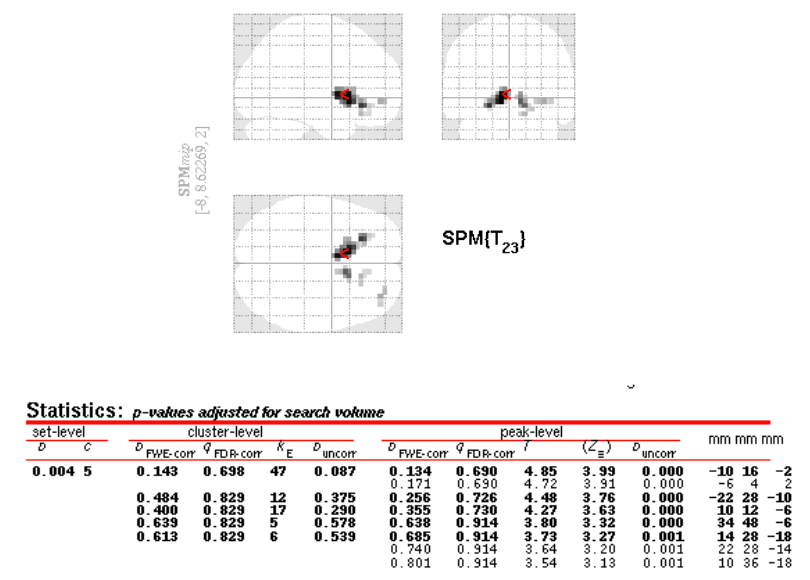

Supplement: Figure S1 — Imaging table and glass brain for DJONES regressor (p<.001, uncorrected k ≥5, n = 24) in experiment 2 ( 6 sec. human partner). (TIF) [file pcbi.1003275.s001.tif]

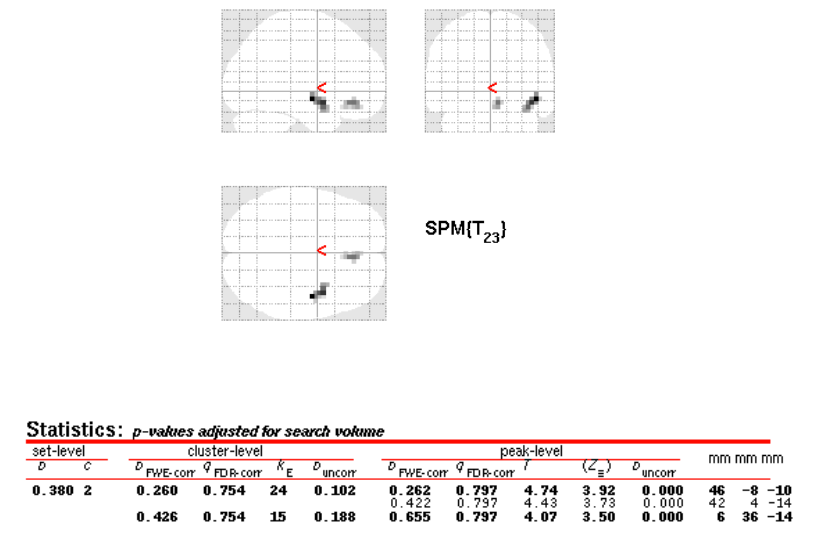

Supplement: Figure S2 — Imaging table and glass brain for DJONES regressor (p<.001, uncorrected k ≥5, n = 24) in experiment 3 ( 6 sec. computer control partner). (TIF) [file pcbi.1003275.s002.tif]

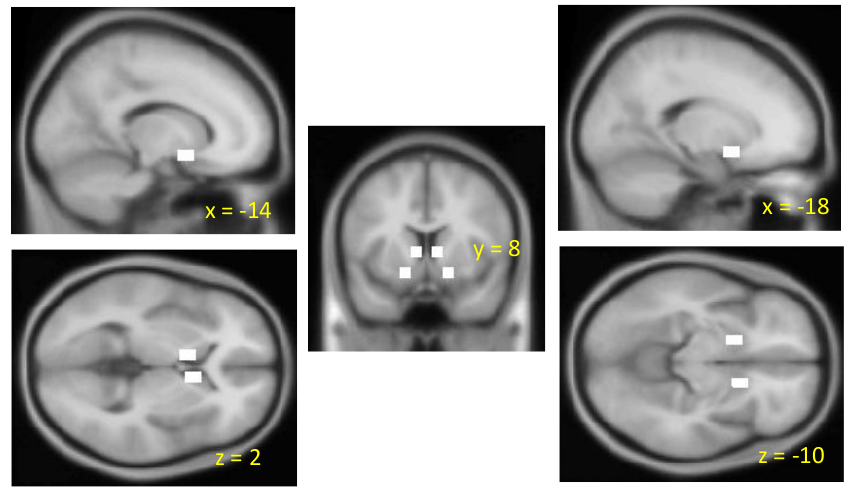

Supplement: Figure S3 — Images of mask used in small volume corrections. Regions of L/R caudate and L/R venral striatum formed by the union of 5 mm radius balls centered on peak activation coordinates from Lohrenz et al. 2007 [24] (Caudate: (-8, 8, 4), (8, 12, 4). Ventral Striatum: (-16, 8,-12), )16, 12,-12). MNI coordinates). (TIF) [file pcbi.1003275.s003.tif]

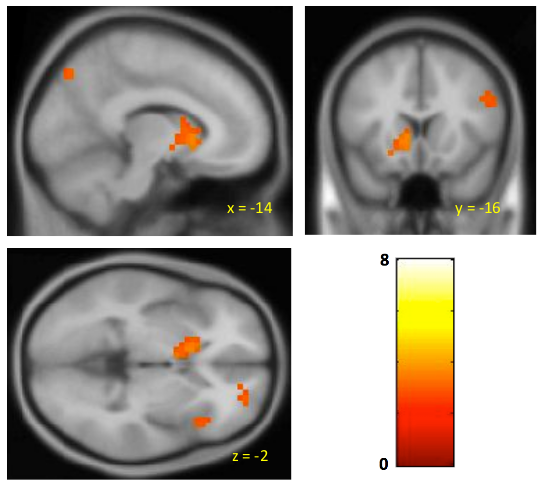

Supplement: Figure S4 — Two-sample t-test image of the comparison human>computer for the DJONES regressor (p<.005. cluster size > = 5, uncorrected, n1 = 24, n2 = 24). (TIF) [file pcbi.1003275.s004.tif]

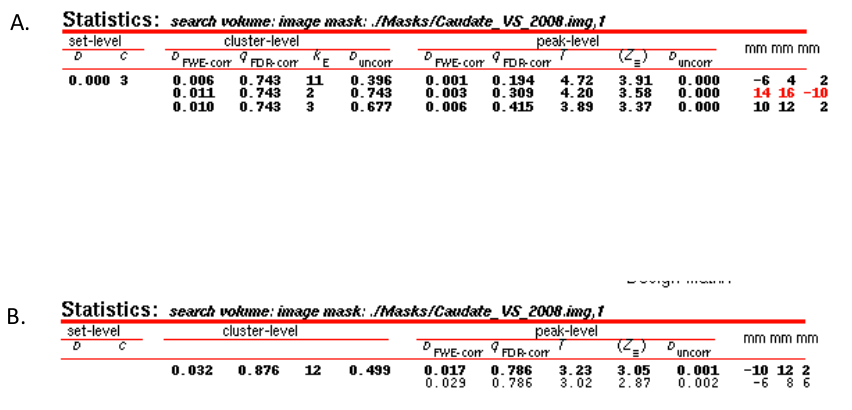

Supplement: Figure S5 — Small volume correction statistics for: A. the DJONES regressor in experiment 2 over the region of interest displayed in Figure S3; B the comparison human > computer (experiment 2 > experiment 3) for the DJONES regressor. (TIF) [file pcbi.1003275.s005.tif]

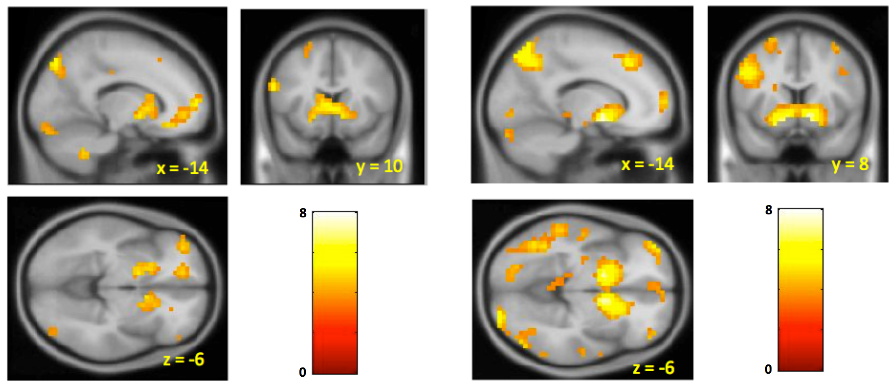

Supplement: Figure S6 — Left: MKT regressor in experiment 2, human partner (p<.001, cluster size > = 5, uncorrected, n = 24); Right: MKT regressor in experiment 3, computer control partner (p<.001, cluster size > = 5, uncorrected, n = 24). (TIF) [file pcbi.1003275.s006.tif]

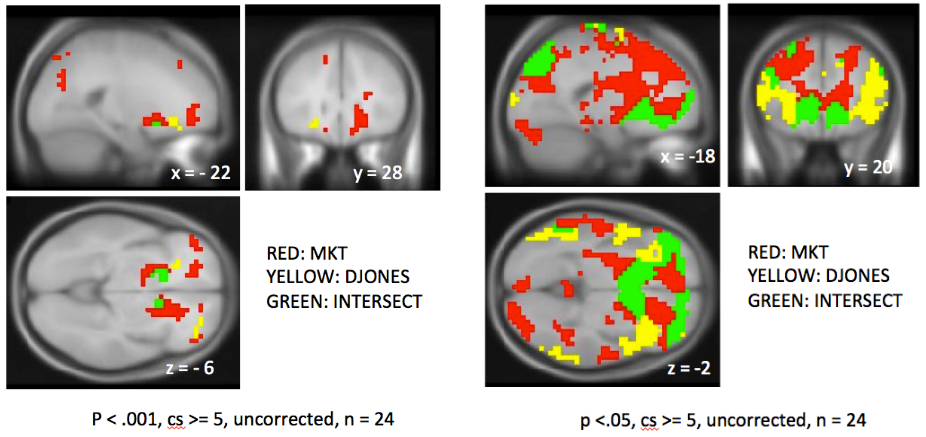

Supplement: Figure S7 — Conjunction/disjunction images for the DJONES and MKT regressors in experiment 2 (human partner). Left: masks created using p<.001, cluster size > = 3, uncorrected, n = 24; Right: masks created using p<.05, cluster size > = 3, uncorrected, n = 24. (TIF) [file pcbi.1003275.s007.tif]

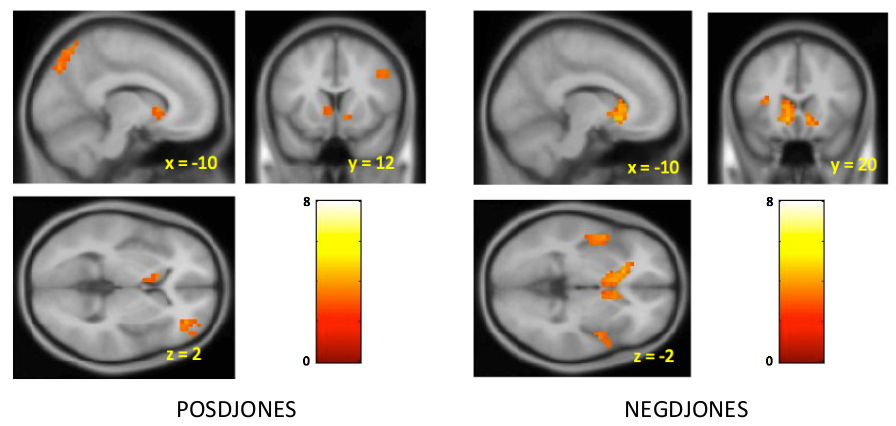

Supplement: Figure S8 — Left: Thresholded t-map of the regressor POSDJONES in experiment 2 (p<.001, cluster size > = 5, uncorrected, n = 24; nb: positive correlation). Right: Thresholded t-map of the regressor NEGDJONES in experiment 2 (p<.001, cluster size > = 5, uncorrected, n = 24; note: negative correlation). (TIF) [file pcbi.1003275.s008.tif]

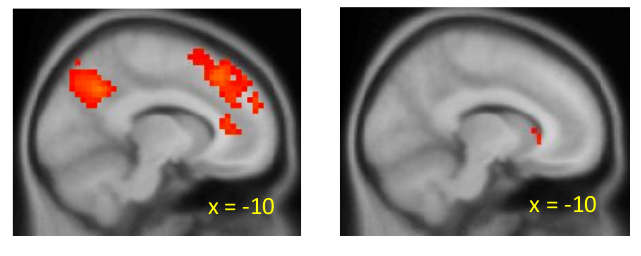

Supplement: Figure S9 — Thresholded t-maps for the within-subject contrast NEGDJONES+POSDJONES. Left, positive correlation, right negative correlation (p<.05, cs > = 5, uncorrected, n = 24). (TIF) [file pcbi.1003275.s009.tif]

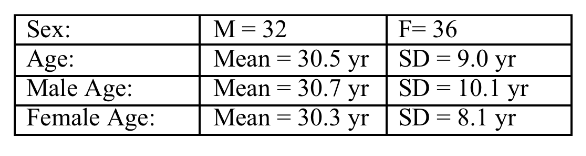

Supplement: Table S5 — Contrast estimates for the grouped three-experiment behavior. (TIF) [file pcbi.1003275.s014.tif]

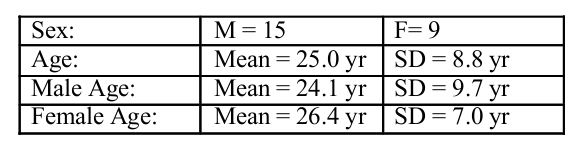

Supplement: Table S6 — Behavioral regression fixed-effect coefficient estimates for experiment 2 (6 sec human partner for the POSDJONES/NEGDJONES model; n = 24). (TIF) [file pcbi.1003275.s015.tif]

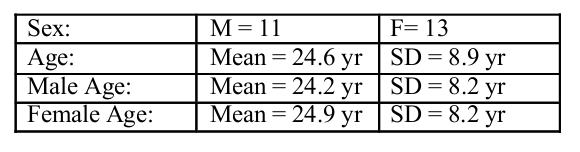

Supplement: Table S7 — Contrast estimate for experiment 2 (6 sec human partner for the POSDJONES/NEGDJONES model; n = 24). (TIF) [file pcbi.1003275.s016.tif]
