# Supplementary material for: Keeping up with the Joneses: Interpersonal Prediction Errors and the Correlation of Behavior in a Tandem Sequential Choice Task
Source: PLoS Comput Biol. 2013 Oct 24;9(10):e1003275. doi: 10.1371/journal.pcbi.1003275 (PMC3812045; doi:10.1371/journal.pcbi.1003275)
Supplement: Text S1 — Supplementary information on task and analysis. (DOCX) [file pcbi.1003275.s017.docx]

*Supplementary Information*

*Participants*

(Experiment 1, 2 sec Jones delay). 76 participants were recruited and 74 scanned in accordance with a protocol approved by the Baylor College of medicine IRB. In the two behavioral only subjects the log files of the experiment were incomplete, leaving unusable data; in two scanned subjects the experiment terminated prematurely; in 4 other scanned subjects the functional images were unusable, leaving 68 subjects with both behavioral and imaging data. Table S1 summarizes the demographic information of these 68 subjects. All data (for the 2 sec cohort) mentioned in the text and Supplementary Information (SI) refers to these 68 subjects.

(Experiment 2, 6 sec Jones delay). 24 participants were recruited and 24 scanned in accordance with a protocol approved by the Virginia Tech IRB.

(Experiment 3, 6 sec Jones delay, computer Controls). 25 participants were recruited and 25 scanned in accordance with a protocol approved by the Virginia Tech IRB. One subject’s data was not included because the experiment concluded prematurely.

*Task Details.* In both experiments participants arrived at the lab, were consented, and then read task instructions. They did not meet. After they were loaded in scanner, the task began. Each subject participated in 10 markets in a random order. There were two groups of markets, A and B (originally described and used in Lohrenz et al. [1] ). After seeing initial market data, a participant selected an investment level (0% to 100% in increments of 5%) using one button box (shown on a slider bar on the screen) and submitted the decision using the other button box. 750 ms after the later of the two partners submitted the next market result was displayed. 2 sec (6 sec for experiments 2 and 3), later two red arrows appeared on each participants screen on either side of the slider bar showing the level of the other person’s investment. The arrows remained on the screen 2 sec, were then removed, the bar grayed out, and then returned to red 750ms later to signal changing the investment was allowed. This was repeated 20 times per market, for a grand total of 200 decisions.

*Scanning Details.*

fMRI data acquisition and analysis: Neural data were collected using 3-tesla Siemens TRIO scanners on the scanning subjects described above. Initial high-resolution T1-weighted scans were acquired using an MP-Rage sequence. Functional images were acquired with TR = 2000 ms and TE = 25 ms. 37 4- mm slices were acquired tilted 30 degrees to the anteroposterior commissural line, yielding functional voxels that were 3.4mm x 3.4mm x 4 mm.

*Preprocessing*: All data were preprocessed using standard SPM8 algorithms (<http://fil.ion.ucl.ac.uk/spm>, ). Functional images were motion corrected using a six-parameter rigid-body transformation to the first functional scan. The mean functional images for each respective subject were then co-registered using a twelve-parameter affine transformation to the subject’s high resolution T1 structural scan. The subject T1 was segmented into gray and white matter and then normalized to the MNI template, and the functional images normalized to the template, with resampled 4x4x4mm functional voxels. Functional images were smoothed spatially using a 8mm Gaussian kernel.

*fMRI Analysis.* All functional data were high-pass (128 sec) filtered. The AR(1) error structure option was used in SPM8. For each subject a design matrix was constructed using the following events (each event was punctate, and convolved with the standard hemodynamic response function (HRF) in SPM2): NEWMARKET (New market display), CLEAR (image removed from screen), INITIALREVEAL (First reveal of a particular market), REVEAL1 (Reveal of first result), REVEAL (reveal of results 2-19), ENDREVEAL (last result), JONESREVEAL (Other person’s bet revealed), JONESOFF (Jones Arrows removed), SUBMIT (result submission), KEYPRESS (other keypresses, including moving the slider bar up and down to select investment, not including submit). The model referred to in the text includes 2 parametric regressors: Mkt “attached” to REVEAL (i.e. defined a new column in the design matrix formed by modulating the punctate event REVEAL by the parameters and then convolving with the HRF), and DJones attached to the punctate event JONESREVEAL. We now define these terms in detail. The basic building block is the market return, defined as

,

whereis the market price at time *t*.

We define the parameter :

(Note that the bet is the fraction of current wealth allocated to the market).

The MKT and DJONES regressor are constructed by modulating the REVEAL and JONESREVEAL regressors by the respective parameters.

The small volume correction (SVC) referred to in the text (for both the DJONES human condition and for the comparison of DJONES across the 6 sec human and control condition) used a mask consisting of the union of 4 5mm balls centered on the peak activation in L/R caudate and L/R ventral striatum taken from [1]. The MNI coordinates of these points are (-8,8,4), (8,12,4), (-16,8,-12), and (16,12,-12).

The across-subject regression in Figure 4 used the subjects’ coefficients of DJONES from the by-subject regression

estimated by the function lmList in R package nlme [2-3]. The neural regression was estimated in SPM8 using a gray-matter mask constructed by setting voxel values equal to 1 if prob(grey matter) >= .4, 0 otherwise.

*Behavioral Analysis.*

For the main behavioral analysis we applied a linear mixed effect model with subject as the grouping variable. The analysis was done using the R function lme in the package nlme [2-3]. The model was given by

where is the z-score (over the experiment) of the investment at time *t.* We included indicator functions for each group (1 = 2 sec human, 2 = 6 sec human, 3 = 6 sec computer) interacting with each independent variable. $RE_{t}$ is the random effects term, defined by

$$RE_{j,\text{ t}}=a_{0,j}+\tilde{b}_{j,\text{ t}}\cdot a_{1,\text{ }j}+MKT_{j,\text{ }t}\cdot a_{2,\text{ }j}+DJONES_{j,\text{ }t}\cdot a_{3,\text{ }j}.$$

For each subject *j* the random effects coefficients $(a_{0,j},a_{1,j},a_{2,j},a_{3,j})$ are assumed to be distributed as multivariate mean zero Gaussian, independent of the error term . We include a table of estimates in Table S3. For the behavioral analysis of POSDJONES and NEGDJONES, we defined

$$\begin{matrix} POSDJONES\text{ } =\max(DJONES,0),\text{ and} \\ NEGDJONES\text{ } =\max(-DJONES,0), \end{matrix}$$

as the positive and negative parts of DJONES. We ran a mixed effects model similar to the above model over the behavior of the 6 sec human group only:

where

$$RE_{j,\text{ t}}=\tilde{b}_{j,\text{ t}}\cdot a_{1,\text{ }j}+MKT_{j,\text{ }t}\cdot a_{2,\text{ }j}+POSDJONES_{j,\text{ }t}\cdot a_{3,\text{ }j}+NEGDJONES_{j,\text{ }t}\cdot a_{4,\text{ }j}$$

are the random effects (note: there are no random effects on the constant term because the model fit (AIC) was better without it).

References

1. Lohrenz T, McCabe K, Camerer CF, Montague PR. (2007) Neural signature of fictive learning signals in a sequential investment task. Proc Natl Acad Sci U S A 104: 9493-9498.

2. R Core Team. (2013) R: A language and environment for statistical computing. R Foundation for Statistical Computing, Vienna, Austria.

3. Pinheiro J, Bates D, DebRoy S, Sarkar D, R Development Core Team (2013). nlme: linear and nonlinear mixed-effects models. R package version 3.1-109.
